# Different Host Complement Systems and Their Interactions with Saliva from *Lutzomyia longipalpis* (Diptera, Psychodidae) and *Leishmania infantum* Promastigotes

**DOI:** 10.1371/journal.pone.0079787

**Published:** 2013-11-08

**Authors:** Antonio Ferreira Mendes-Sousa, Alexandre Alves Sousa Nascimento, Daniel Costa Queiroz, Vladimir Fazito Vale, Ricardo Toshio Fujiwara, Ricardo Nascimento Araújo, Marcos Horácio Pereira, Nelder Figueiredo Gontijo

**Affiliations:** Department of Parasitology, Universidade Federal de Minas Gerais, Belo Horizonte, Minas Gerais, Brazil; Louisiana State University, United States of America

## Abstract

**Background:**

*Lutzomyia longipalpis* is the vector of *Leishmania infantum* in the New World, and its saliva inhibits classical and alternative human complement system pathways. This inhibition is important in protecting the insect´s midgut from damage by the complement. *L. longipalpis* is a promiscuous blood feeder and must be protected against its host’s complement. The objective of this study was to investigate the action of salivary complement inhibitors on the sera of different host species, such as dogs, guinea pigs, rats and chickens, at a pH of 7.4 (normal blood pH) and 8.15 (the midgut pH immediately after a blood meal). We also investigated the role of the chicken complement system in *Leishmania* clearance in the presence and absence of vector saliva.

**Results:**

The saliva was capable of inhibiting classical pathways in dogs, guinea pigs and rats at both pHs. The alternative pathway was not inhibited except in dogs at a pH of 8.15. The chicken classical pathway was inhibited only by high concentrations of saliva and it was better inhibited by the midgut contents of sand flies. Neither the saliva nor the midgut contents had any effect on the avian alternative pathway. Fowl sera killed *L. infantum* promastigotes, even at a low concentration (2%), and the addition of *L. longipalpis* saliva did not protect the parasites. The high body temperature of chickens (40°C) had no effect on *Leishmania* viability during our assays.

**Conclusion:**

Salivary inhibitors act in a species-specific manner. It is important to determine their effects in the natural hosts of *Leishmania infantum* because they act on canid and rodent complements but not on chickens (which do not harbour the parasite). Moreover, we concluded that the avian complement system is the probable mechanism through which chickens eliminate *Leishmania* and that their high body temperature does not influence this parasite.

## Introduction

American visceral leishmaniasis (AVL), caused by *Leishmania infantum* (syn. *L. chagasi*) (Kinetoplastida; Trypanosomatidae), is a life-threatening disease in humans that is distributed throughout Latin America, from Mexico to Argentina, with 90% of cases occurring in Brazil [1]. It is transmitted by the bite of infected female sand flies, principally *Lutzomyia longipalpis* (Diptera, Psychodidae). Like other *Leishmania* species in the New World, *L. infantum* has a zoonotic cycle with complex epidemiology, and it parasitises different species of mammals, including humans, canids, opossums and rodents [2]. 

Domestic dogs are considered to be the most significant parasite reservoirs because they present intense cutaneous parasitism, thus providing an easy infection source to the vector, and they also live closer to humans than sylvatic reservoirs such as wild canids of the genus *Cerdocyon* [3]. In addition, the prevalence of AVL in dogs is greater than in humans [4]. Once infected, humans are not considered good reservoirs because they usually have few skin parasites. The role of rodents as potential reservoirs is still under discussion. If confirmed, rodents could represent a risk for parasite transmission in the peridomicile since some of them have developed synanthropic habits [5]. 

The role of domestic chickens in AVL epidemiology is not yet completely understood [6,7]. Although they are not suitable hosts for *Leishmania*, chickens are common blood sources for *L. longipalpis*, and their coops are known to be resting places for sand flies, which makes poultry very important in the maintenance of vector populations [6]. Studies have suggested that the presence of domestic chickens could be a risk factor for acquiring AVL in endemic areas [7,8]. Conversely, it was also proposed that chickens could act as a zooprophylaxis for AVL [6] because they are refractory to leishmaniasis, with the ability to kill parasites transmitted by infected vectors through unknown mechanisms [9]. Their high body temperature (over 40°C) and potent complement system could be the causes of their parasite incompatibility, but no studies have investigated these hypotheses to our knowledge [6,10].

The vector regurgitates infective promastigote parasites from its intestines and the contents of its salivary glands into the host skin during a blood meal [11]. *L. longipalpis* adult females present a pair of salivary glands and, as do several other haematophagous arthropods, exudes a repertoire of pharmacologically active molecules in its saliva that facilitate blood feeding and parasite transmission. Amongst these molecules are (at least) a vasodilator, an anti-clotting compound and an anti-platelet aggregation factor [12,13,14]. Host immune system modulators are also present [14,15]. Amongst these molecules, there are inhibitors of the human complement system's classical and alternative pathway [16]. Recent studies have demonstrated that complement inhibitors are present in the saliva, in the midgut or even in both places in various haematophagous arthropods [17,18,19,20].

The complement system is triggered by the following three distinct pathways: the classical pathway, which is activated by antibody-antigen complexes; the lectin pathway, which is activated by a mannose binding lectin; and the alternative pathway, which is activated by non-self surfaces, such as those of pathogens. These three pathways converge to a common point where the activation of C3 molecules occurs through a proteolytic cleavage promoted by the C3 convertases, producing C3b molecules that bind covalently to the activator surface. These molecules subsequently promote the assembly of the membrane attack complex (MAC), which is responsible for membrane lysis [21]. 

The presence of complement inhibitors in haematophagous arthropods is very important because these inhibitors are involved in protecting the midgut epithelium against complement attack [18]. Because *L. longipalpis* feeds on various vertebrate species, which exposes them to the potential deleterious effects of vertebrate complement systems, it is important to understand how the vector addresses this challenge. Here, we report our investigations about *L. longipalpis* saliva effects on the complement system of dogs, guinea pigs, rats and chickens. Because the chicken complement system was not efficiently inhibited by *L. longipalpis* saliva, the vector's intestinal contents were examined for inhibitors. These assays were performed at pH levels of 7.4 (the normal pH of circulating blood) and 8.15 (the typical phlebotomine midgut pH just after a blood meal [22]). The role of chicken complement in eliminating *L. infantum* promastigotes was also investigated. A discussion about the possible role of complement inhibitors in *Leishmania* transmission is provided.

## Methods

### Ethics Statement

The experimental procedures used in this study were approved by the Ethics Committee in Animal Experimentation at the Universidade Federal de Minas Gerais (CETEA/UFMG) under study number 087/11.

### Sand fly maintenance, saliva preparation and intestinal contents

The *L. longipalpis* sand flies in this study originated from Teresina, in the Brazilian state of Piauí. The insects were reared in the Laboratório de Fisiologia de Insetos Hematófagos at the Universidade Federal de Minas Gerais in Brazil using existing methodology [23]. Salivary glands from 4- to 8-day-old unfed females were dissected in saline solution (0.9% NaCl), and their glands were transferred to microcentrifuge tubes on ice. The glands were pooled and stored in 0.9% saline solution for the haemolytic assays. ELISA-like assays designed to detect C3b deposition in classical and alternative pathways were used, for which the glands were stored in HNCM (4 mM HEPES, 145 mM NaCl, 2 mM CaCl_2_, 1 mM MgCl_2_, pH 7.4) or in HNEBM solution (5 mM HEPES, 140 mM NaCl, 10 mM EGTA, 5 mg/ml BSA, 7 mM MgCl_2_, pH 7.4), respectively. The pooled glands were then sonicated for 30 s and centrifuged at 10,000 g for 10 min at 4°C. 

To collect the intestinal soluble contents, groups of 8 midguts from unfed females were dissected and transferred to 12.5 µl of 0.9% saline on a glass slide, where they were opened with needles and their contents were washed. The soluble contents were then transferred to a tube on ice and centrifuged as described for the salivary glands. 

For each assay, the glands or intestinal contents were collected for immediate use or stored at -70°C to be used on the next day. Protein concentration in saliva and intestinal content from 4- to 8-day-old unfed females were measured by the Bradford method [24]. The concentration of salivary protein was 0.22 (±0,02) µg per gland and 1.1 (±0,09) µg per midgut soluble content.

### Sources of sera

Blood samples were collected from healthy dogs (*Canis familiaris*), guinea pigs (*Cavia porcellus*), rats (*Rattus norvegicus*) and chickens (*Gallus gallus*). The blood samples were clotted at 37°C for 1 h and centrifuged at 5,000 g for 5 min at 4°C for serum separation. A pool of sera from each species was aliquoted and stored at -70°C. Each aliquot was used only once. We used fresh chicken sera for flow cytometry assays, and the blood was collected and tested on the same day. 

### Parasites

Axenic *L. infantum* (MHOM/BR/1972/BH46) promastigotes were cultured in Schneider's Insect Medium (Sigma-Aldrich, code S-9895, St. Louis, MO, USA) at a pH of 7.0 supplemented with 10% heat-inactivated foetal calf serum, 100 U/ml penicillin and 100 µg/ml streptomycin. The cultures were incubated at 24±1°C in a B.O.D. incubator. Stationary phase promastigotes were harvested by centrifugation (1,100 g at 20°C for 10 min) and washed twice in 40 mM HEPES, 0.7% NaCl buffer, pH 7.4. The cells were counted in a Neubauer chamber and diluted with the same solution to concentrations of 2 x 10^6^ or 4 x 10^6^ cells/ml according to the assay of interest. 

### Haemolytic assays

To detect the effect of saliva on the classical pathway, we performed haemolytic assays using antibody-sensitised sheep red blood cells. Firstly, 500 µl of sheep erythrocyte suspension (conserved in Alsever's solution) were washed 3 times by centrifugation at 480 g for 5 min at 4°C in 5 ml of GHB-EDTA solution (5 mM HEPES, 145 mM NaCl, 10 mM EDTA and 0.1% gelatin, pH 7.4). After the last wash, the cells were resuspended in 3 ml of GHB-EDTA, then 3 µl of rabbit anti-sheep erythrocyte serum were added and the tube was incubated at 37°C under gentle agitation for 30 min for antibody opsonisation. After incubation, the cells were washed once in GHB-EDTA and twice in GHB^2+^ solution (5 mM HEPES, 145 mM NaCl, 0.15 mM CaCl_2_, 0.5 mM MgCl_2_ and 0.1% gelatin, pH 7.4). The cell concentration was adjusted to 2 x 10^8^ cells/ml before each experiment. 

For the assays, 25 µl of saliva solution at the desired concentration were added to 50 µl of diluted sera in microcentrifuge tubes. Sera were diluted in GHB^2+^ solution at a sufficient concentration to lyse approximately 90% of the erythrocytes. Dog, guinea pig, rat and chicken sera were diluted by 1:40, 1:200, 1:100 and 1:30, respectively. Afterwards, 50 µl of sensitised erythrocytes were added, and the tubes were incubated at 37°C for 30 min. Following incubation, 500 µl of cold saline (0.9% NaCl) were added to stop the haemolytic reaction. The tubes were then centrifuged at 1,700 g for 30 s, and 200 µl of the supernatant were transferred to a 96-well microplate and read at 415 nm in a microplate reader (Molecular Devices, Sunnyvale, CA, USA).

The haemolytic assays were carried out in duplicate, and three controls were used in each test: total haemolysis, in which 500 µl of cold distilled water were added instead of cold saline; spontaneous haemolysis, in which sera were substituted by the same volume of GHB^2+^; and a positive control, in which sera were added without saliva.

Rabbit erythrocytes were used for alternative pathway assays. These cells were not opsonised with antibody. Before the experiments, 500 µl of rabbit blood were washed 3 times in 5 ml of Mg-EGTA solution (5 mM HEPES, 145 mM NaCl, 10 mM EGTA, 7 mM MgCl_2_ and 0.1% gelatin, pH 7.4). The erythrocyte concentration was adjusted to 1 x 10^8^ cells/ml and the sera from dogs, guinea pigs, rats and chickens were diluted by 1:5, 1:5, 1:2 and 1:10, respectively (the serum concentrations were just high enough to lyse approximately 90% of the erythrocytes). Alternative pathway triggering in rodents was not particularly effective. As a consequence, despite the high concentration of serum (1:5 for guinea pigs and 1:2 for rats), the maximal haemolysis percentages for those two animals were 48.1 ± 2.1 and 29.3 ± 3.5, respectively. The rest of the experiment was performed as described for the classical pathway.

The experiments were all repeated with erythrocytes and sera diluted in solutions with a pH of 8.15 (in GHB^2+^ solution for the classical pathway and Mg-EGTA solution for the alternative pathway). Assays at a pH of 8.15 were carried out for both pathways and were performed without saliva to evaluate whether the alkalinisation led to complement inhibition by itself. 

Haemolytic assays with the intestinal contents were carried out with chicken sera only. Twenty-five microlitres of diluted sera (at 1:20 in GHB^2+^ solution for the classical pathway and at 1:10 in Mg-EGTA solution for the alternative pathway) were pre-mixed with 12.5 µl of intestinal content at the desired concentrations (with amounts equivalent to 1, 2, 4 or 8 midguts). To this mixture, 25 µl of sensitised sheep red blood cell suspension (for the classical pathway) or non-sensitised rabbit blood cell suspension (for the alternative pathway) were added. The rest of the assay was performed as described above, using the same controls.

### ELISA-like assays for C3b detection

 To detect the C3b in the classical pathway, a 96-well ELISA plate (COSTAR, code 9017, Corning, NY, USA) was sensitised overnight inside a humid chamber with 50 µl of carbonate/bicarbonate buffer (35 mM Na_2_CO_3_, 15 mM NaHCO_3_, pH 9.6) containing 2 µg of IgG purified from dogs or guinea pigs. The antibodies (IgG) were purified by affinity chromatography using Protein-A Sepharose [25]. 

 Following the plate sensitisation, the wells were blocked with 200 µl of TNB solution (10 mM Tris, 140 mM NaCl and 3% BSA, pH 7.4), followed by a second block with 200 µl of TNBTC solution (10 mM Tris, 140 mM NaCl, 3% BSA, 0.05% Tween-20 and 5 mM CaCl_2_, pH 7.4), both during 30 min under constant agitation at room temperature. Twenty microlitres of 1:20 serum diluted in HNCM solution were added after being premixed in a microcentrifuge tube with 80 µl of HNCM containing different concentrations of saliva, for a total of 100 µl of mixture per well. The plate was then incubated for 30 min at 37°C under agitation (160 rpm). Wells without saliva and without serum were used as positive and negative controls, respectively. 

 The activation of the classical pathway by plate surface-adhered IgG promotes the covalent binding of C3b molecules to the plate surface. Bound C3b can be quantified by specific antibodies. After sera incubation, the wells were washed twice with 200 µl of washing buffer (10 mM Tris, 140 mM NaCl and 0.1% BSA, pH 7.4) for 2 min at room temperature under agitation (160 rpm). Fifty microlitres of anti-C3 antibody diluted in HN solution (10 mM HEPES and 140 mM NaCl, pH 7.4) were added to the wells, and the plate was incubated for 30 min under agitation (160 rpm) at room temperature. The goat antibodies anti-dog C3 (ICL, code GC3-401, Portland, OR, USA) and anti-guinea pig C3 (ICL, code GC3-601-Z, Portland, OR, USA) were diluted in HN solution to 1:3,000 and 1:1,000, respectively. The wells were washed twice as before, and 50 µl of anti-goat antibody conjugated with peroxidase (Calbiochem, code 401504, La Jolla, CA, USA) diluted at 1:1,500 in HN solution were added to the plate, which was incubated again for 30 min at room temperature under agitation. After two washes, the wells were filled with 200 µl of developing buffer (50 mM sodium citrate, 50 mM Na_2_HPO_4_, 1 mg/ml o-phenylenediamine (Sigma, code P-9029, St Louis, MO, USA) and 0.075 % H_2_O_2_ (Synth, code P2233.01.BJ, Diadema, SP, Brazil), pH 5.0). The plate was read in a microplate reader (Molecular Devices, Sunnyvale, CA, USA) at 450 nm and 37°C for 10 min in the kinetic mode (one read every 30 s). The maximal velocities (rate of absorbance increase) of the reactions were calculated with SoftMax Pro 5.2 software, and the resulting data were used for statistical analysis. 

 For assays of the alternative pathway, the wells were covered with 100 µl of agarose 0.1% solution and allowed to dry overnight at 37°C. Twenty microlitres of sera diluted by 1:3 (dog sera) and 1:5 (guinea pig sera) in HNEBM solution were added to the wells in addition to different quantities of saliva diluted in the same solution, for a total of 100 µl per well. The plate washes and antibody dilutions were completed with HNB solution (10 mM HEPES, 140 mM NaCl and 1% BSA, pH 7.4). All of the other steps were performed as described for the classical pathway assays. 

 Assays were also performed for both species at a pH of 8.15. All of the assays were performed in triplicate, with at least three independent repetitions. C3b deposition assays were not performed with chicken and rat sera due to a lack of anti-C3 antibodies for these species. 

### Real-time kinetics of *L. infantum* promastigote lysis

 To evaluate the role of the complement system in *L. infantum* clearance by chickens, real-time kinetic assays were performed in a flow cytometer (BD FACScan, Franklin Lakes, NJ, USA) with fresh chicken sera. Ten microlitres of propidium iodide (PI) (Sigma, code P-4170, St Louis, MO, USA) solution (0.01 mg/ml) were mixed with 100 µl of *Leishmania* suspension containing 2 x 10^6^ promastigotes in a microcentrifuge tube. The PI is a DNA intercalating dye that binds to the DNA of dead cells and emits strong fluorescence when excited. For the assay, 90 µl of chicken sera at different concentrations (2%, 5% and 10%) were added to the tubes containing the promastigotes (final volume 200 µl). The mixture was incubated at 37°C in a water bath for 12 min. Aliquots of 20 µl were collected at different intervals (30 s, 3 min, 6 min, 9 min and 12 min), transferred to FACS tubes containing 180 µl of 0.9% saline and ran on the flow cytometer using BD CellQuest Pro software for cell counting until approximately 15,000 events had occurred. 

 To investigate whether the vector saliva could protect the parasites from chicken complement system, we employed the methodology described above but mixed 50 µl of 40 mM HEPES, 0.7% NaCl buffer, pH 7.4 with 8 salivary glands and 50 µl of *Leishmania* suspension (4 x 10^6^ promastigotes). The final concentrations of chicken sera were 2%, 5% and 10%. For the controls, assays were performed with preparations without sera or containing inactivated sera (pre-incubated at 56°C for 30 min).

 To determine whether the chicken body temperature had any effect on promastigotes, we incubated promastigotes at 40°C without sera for 12 min in the presence of PI. Cell death was evaluated in the flow cytometer as described. Each experiment was repeated independently at least three times. 

### Statistical analysis

 All statistical tests and graphs were performed using GraphPad Prism version 5 (GraphPad Software Inc.). The means of duplicates (haemolytic assays) and triplicates (ELISA-like assays) were calculated for each independent experiment, and the means of respective negative controls were subtracted. The results were transformed into a percentage of haemolysis inhibition or inhibition of C3b deposition, with the positive control considered to have 100% activity.

Data normality was assessed using the Kolmogorov-Smirnov test. A paired t-test (for two groups) and one-way ANOVA followed by Tukey's test (for more than two groups) were used to analyse normally distributed variables. A statistical analysis of kinetic lysis assays was performed by comparing the results at minute 12. Significance was determined at p < 0.01.

## Results and Discussion

 The complement system is a major component of the vertebrate innate immune response, and it consists of approximately 30 proteins soluble in the serum or associated with cell membranes [21]. The pH of normal human or domestic animal blood is maintained at approximately 7.4; therefore, this is the natural pH expected for complement function in these animals. However, it was demonstrated that the pH in the abdominal midgut of *L. longipalpis* females increases from a pH of 6 to a pH of 8.15 immediately after a bloodmeal in hamsters [22]. If any complement action can be expected inside the midgut of the insect, it most likely occurs at a pH of 8.15. Consequently, the capacities of the classical and alternative pathways were investigated at pHs of both 7.4 and 8.15 for the four species of interest. 

**Figure 1:**
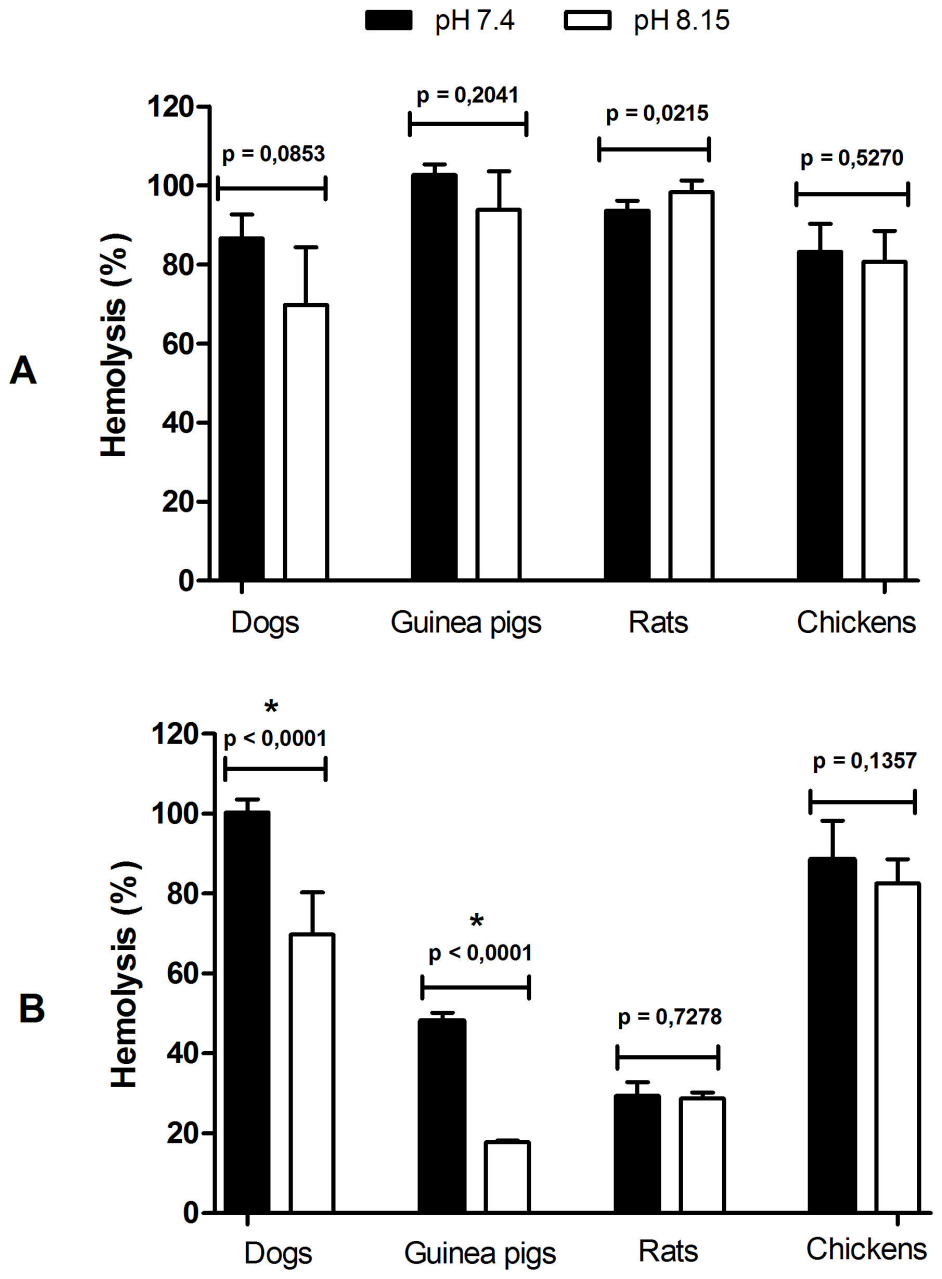
Influence of pH in the activation of complement system from different hosts. Activation of classical (A) and alternative (B) pathways from the serum of dogs, guinea pigs, rats and chickens at pH 7.4 and 8.15.

 According to the results presented in Figure 1A, the functioning of the classical pathway of all four species was similar at a pH of 7.4 as well as at pH 8.15. Conversely, the alternative pathway of dogs and guinea pigs had significantly lower activities at a pH of 8.15 (p<0.0001) when the lytic activity is compared to that of pH 7.4 (Figure 1B). 

 The different serum concentrations used to obtain approximately 90% haemolysis at a pH of 7.4 (or even at pH 8.15) reflected the differences in the classical pathway activity of the species in question. The same could be said for the alternative pathway, but in this case, it was not possible to obtain higher percentages of haemolysis using guinea pig and rat sera, even with lower dilutions such as 1:5 or 1:2, respectively (Figure 1B). The differences observed here could partly be attributed to differences in the triggering capability of the alternative pathway by rabbit erythrocytes (see the material and methods section concerning haemolytic assays for the alternative pathway). 

 The classical pathway results at the physiological pHs encountered in *L. longipalpis* (Figure 1A) led us to believe that this pathway could be triggered inside the vector midgut by binding non-specific antibodies to epithelial cells of the midgut, which represents a risk for the insect. Additionally, the alternative pathway could be triggered by complex carbohydrates usually present in the glycocalyx of the enterocytes, also representing a potential risk to the insect. 

**Figure 2:**
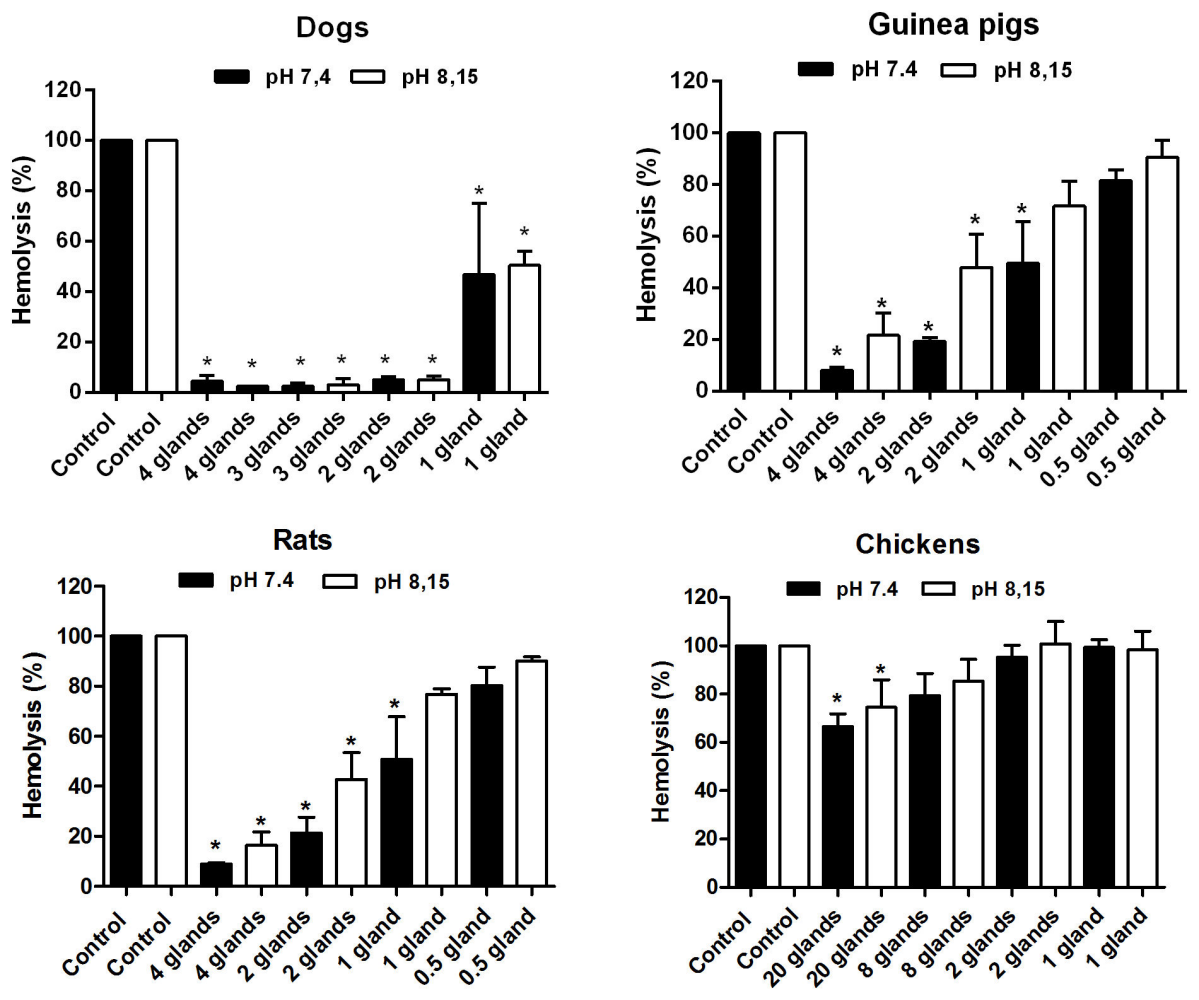
Effect of *L. longipalpis* saliva on the classical pathway of the complement system from different hosts at pH 7.4 and pH 8.15.

 Considering the deleterious potential of the complement to midgut cells, we expected that *L. longipalpis* females would be able to inhibit the complement system of their usual hosts. Accordingly, *L. longipalpis* saliva strongly inhibited the haemolytic activity of the classical pathway of dogs, guinea pigs and rats at both pHs of 7.4 and 8.15 (P < 0.0001) (Figure 2). A similar inhibition pattern was already described for the human classical pathway by our group [16]. The efficiency of classical pathway inhibition suggests that the salivary inhibitor ingested together with the blood of the hosts may be capable of protecting the insect midgut epithelium against the complement system of those mammals mentioned above. 

The results are expressed as the mean of percentage of haemolysis ± SD. Three replicates were performed for each experiment. 

In contrast to the mammals studied here, the chicken classical pathway was only inhibited by high concentrations of saliva (saliva equivalent to 8 and 20 glands) (Figure 2). In this case, it is very probable that the amount of saliva released at the bite site would not be sufficient to protect the intestinal epithelium. Because chickens are a common blood source for *L. longipalpis* [6,26,27], this vector should have other mechanisms to evade the avian complement system and preserve its intestinal cells. 

**Figure 3:**
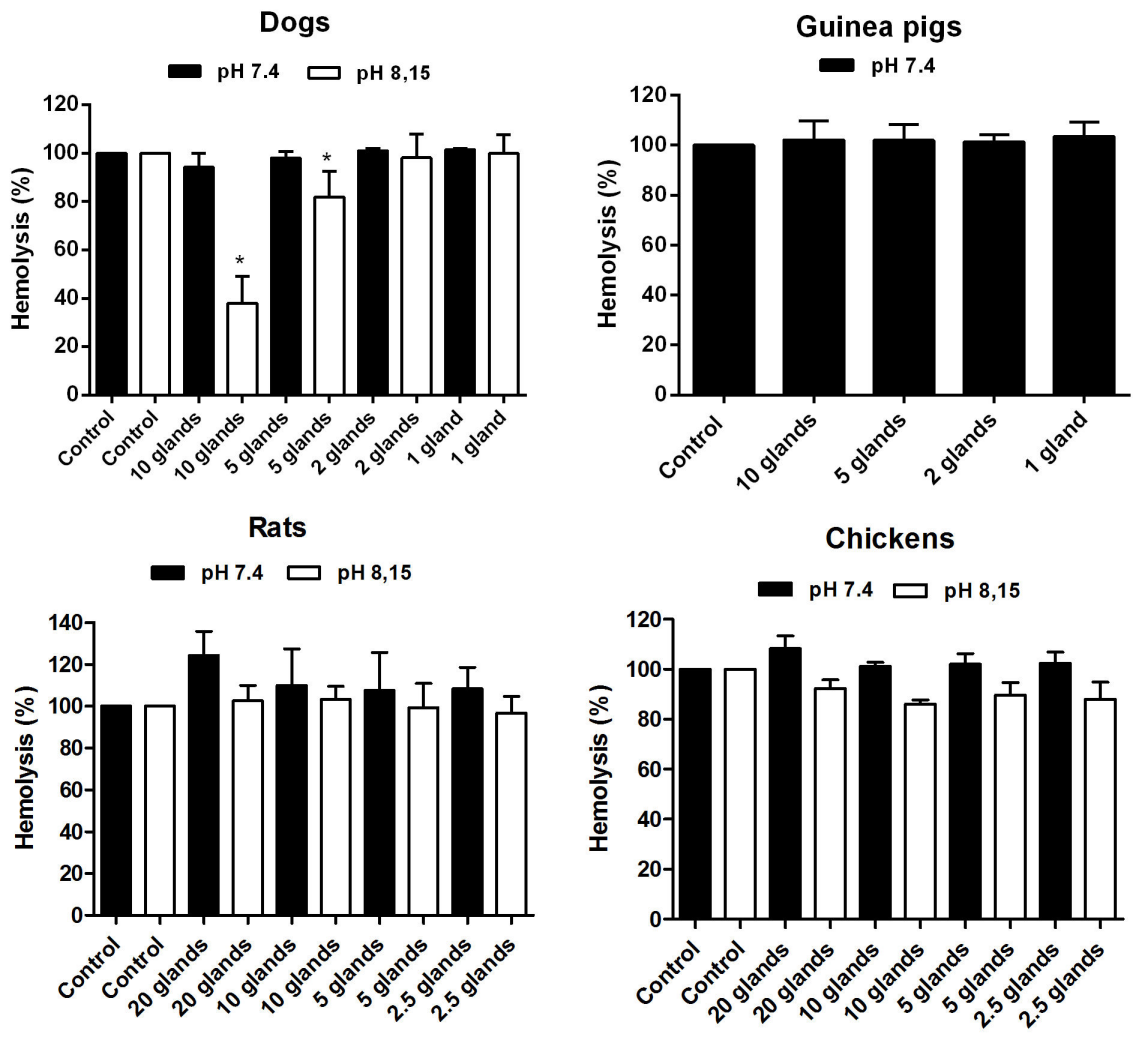
Effect of *L. longipalpis* saliva on the complement alternative pathway of different hosts at pH 7.4 and pH 8.15. The guinea pig alternative pathway could not be assessed at pH 8.15 because the alkalinisation itself affects the pathway's efficiency in a way that the difference between the positive and negative controls is undetectable. The results are expressed as the mean of percentage of haemolysis ±SD. Three replicates were performed for the experiments.

The effect of *L. longipalpis* saliva on the alternative pathway was also investigated. The solution used do dilute sera and rabbit erythrocytes in those experiments contained EGTA, which chelates Ca^2+^ ions necessary for triggering the classical and lectin pathways. This way, only the alternative pathway was activated. With the exception of dog samples at a pH of 8.15, which showed a slight inhibition, the alternative pathways of the other species were not inhibited (Figure 3). These data contrast previous findings [16], which demonstrated the inhibition of the human alternative pathway by *L. longipalpis* saliva at a pH of 7.4. 

**Figure 4:**
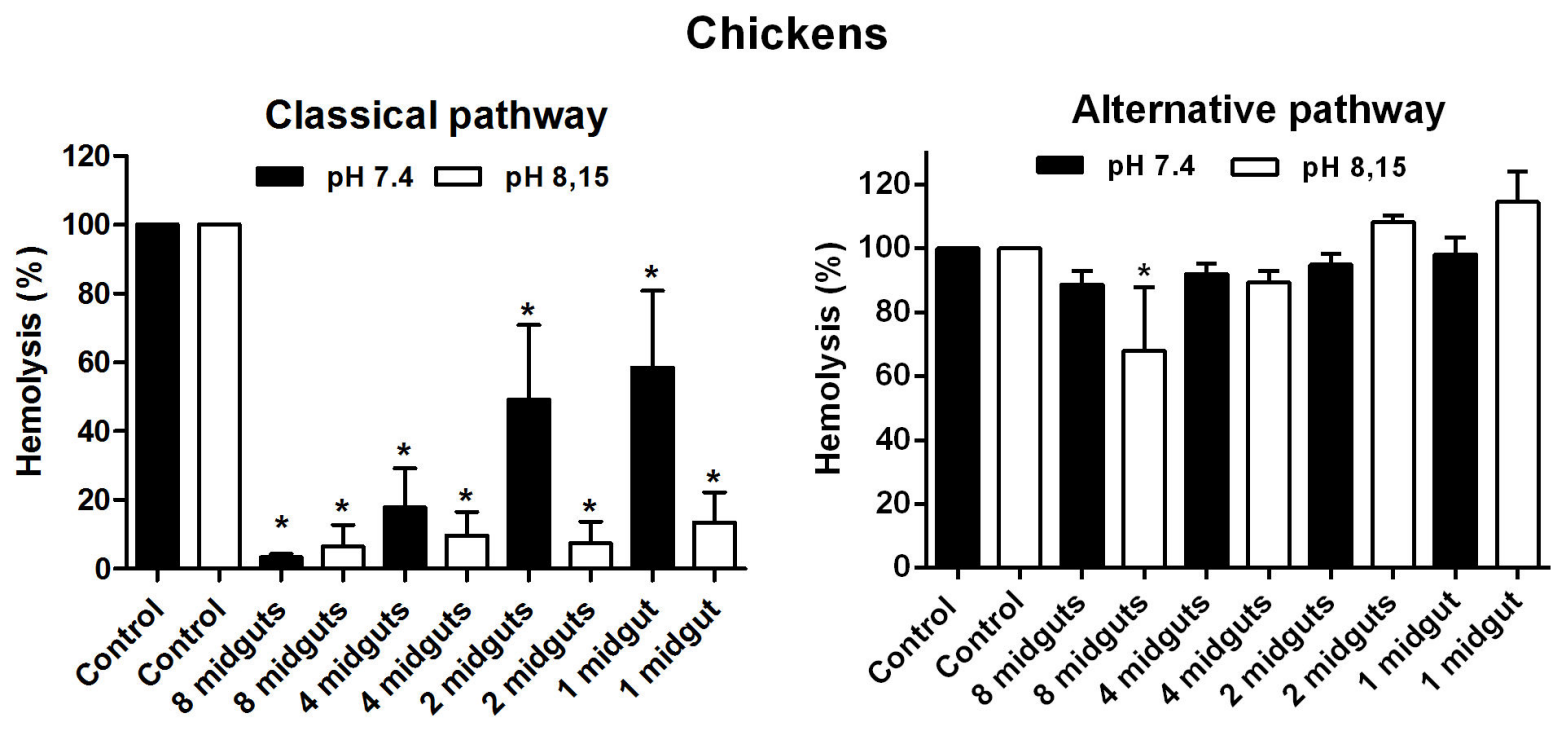
Effect of *L. longipalpis* intestinal content on the classical and alternative complement pathways of chickens at pHs of 7.4 and 8.15. The results are expressed as the mean of percentage of haemolysis ±SD. Three replicates were performed for each experiment.

Our group recently demonstrated the presence of complement inhibitors in the intestinal contents of three species of triatomine bugs as well as in the intestinal content of the mosquito *Aedes aegypti* [18]. Considering the possibility that intestinal inhibitors could be effective against chicken complement (and even against the complement of other hosts), we investigated the luminal content of *L. longipalpis* as a potential source of inhibitors. In fact, the luminal content was efficient in inhibiting the classical pathway of the chickens at both pHs of 7.4 and 8.15 (Figure 4). The alternative pathway was only inhibited at a pH of 8.15 (Figure 4), though a large sample quantity was required (equivalent to 8 midguts). It is important to highlight that the alkalinisation of the midgut after a bloodmeal by itself reduces the alternative pathway activity of dogs, guinea pigs and most likely other species.

 The presence of efficient inhibitors against the classical pathway and the apparent inefficiency or absence of alternative pathway inhibitors suggests that the principal challenge for the insect is the classical pathway. Although not investigated in the present study, it is possible that membrane-bound complement regulators, which are similar to those encountered in mammals such as CD46, DAF, CD59 and others [21,28], could be present in enterocyte microvilli and could participate in epithelium protection, especially against the alternative pathway. Another possibility not investigated here is that salivary and intestinal inhibitors could work synergistically with each other for a more effective inhibition of the classical and alternative pathways. 

Salivary complement inhibitors with species-specific effects, such as those described here, have already been described for three species of ticks [29]. *Ixodes ricinus*, the vector of Lyme disease in Europe, is considered to be as highly promiscuous a bloodfeeder as *L. longipalpis* and has been known to parasitise more than 65 vertebrate species [30]. Its saliva is capable of inhibiting only the alternative pathway of hosts, including humans, deer and hedgehogs, but it causes no effect on the complement of dogs and pheasants. The saliva of *I. hexagonus* was capable of inhibiting the alternative pathway of humans, deer, dogs and hedgehogs, but it did not inhibit the alternative pathway of pigeons and pheasants. Finally, *I. uriae* saliva inhibited the pigeon alternative pathway but showed no effect when tested with human and pheasant sera [29]. Our data suggest that *L. longipalpis* salivary inhibitors follow a species-specific trend. Unfortunately, there is scarce information about the structural and functional differences as well as differences in plasma concentrations of complement components in different animal species.

Amongst the arthropods, the salivary complement inhibitors of ticks are the most studied, especially those belonging to the genus *Ixodes*. It was demonstrated that *I. ricinus* salivary gland extract inhibits the alternative pathway by acting on the α-chain of the C3 component so it could not participate in the convertase assembly, thus leading to a reduced affinity for factor B [31]. Daix et al. [32] cloned and expressed anticomplement proteins from *I. ricinus*, which were denominated IRAC I and IRAC II, and Schroeder et al. [33] demonstrated that these inhibitors act broadly and complementary over the alternative pathway of different host species. Later, Couvreur et al. [17] discovered five more anticomplement proteins in *I. ricinus* saliva with a different mechanism of inhibition. These new inhibitors bind to properdin, leading to the inhibition of C3 convertase in the alternative pathway. Studies with salivary complement inhibitors in other tick species have also been completed, highlighting their importance in local inflammation control and pathogen transmission [34,35].

**Figure 5:**
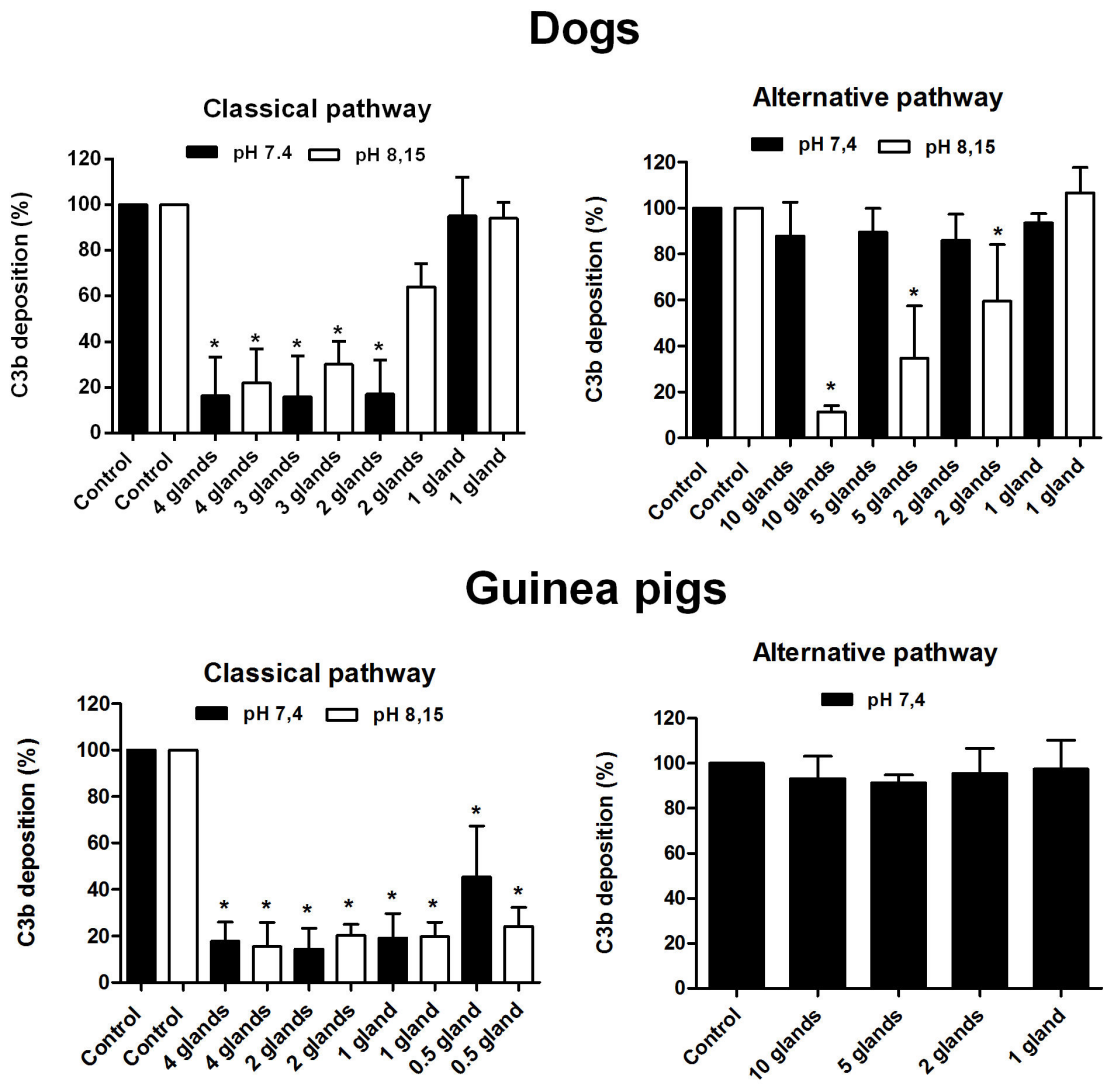
Effect of *L. longipalpis* saliva on the C3b deposition of classical and alternative complement pathways of dogs and guinea pigs at pHs of 7.4 and 8.15. The guinea pig alternative pathway could not be assessed at a pH of 8.15 because the alkalinisation itself affects the pathway's efficiency in a way that the difference between the positive and negative controls is undetectable. The results are expressed as the mean of percentage of C3b deposition ± SD. Three replicates were performed per experiment.

Our C3b deposition assay results confirm those of the haemolytic assays. C3b deposition in dogs and guinea pigs by the classical pathway was inhibited in a dose-dependent manner at both pHs (Figure 5). The C3b deposition in dog alternative pathway was significantly inhibited only at a pH of 8.15 (P < 0.0001). In guinea pigs, the alternative pathway was tested only at a pH of 7.4 because the alkalinisation inhibited C3b deposition by itself (data not shown). 

The inhibition of C3b deposition suggests that the salivary inhibitor (or inhibitors) acts at the level of the C3 component or at a preceding point in the complement cascade. The salivary inhibitor, by acting at the beginning of the classical pathway cascade, in addition to protect the vector midgut, is also responsible for down-regulation of the inflammation at the bite site. This inhibitor may prevent C3 and C5 cleavage and the generation of C3a and C5a subproducts, which are efficient anaphylatoxins that are responsible for recruiting leucocytes to the bite site [21]. Thus, the perception of the vector by the host is diminished, enhancing the chance of a successful blood meal [19]. Moreover, C3a is capable of inducing platelet activation and aggregation, leading to blood clotting [36]. By blocking the normal flow of the classical pathway cascade and consequently blocking C3a generation, the salivary inhibitor may act indirectly as an anti-clotting protein, thus retarding hemostasis and favouring blood flow through the vector mouthparts.

The inhibition of the lectin pathway was not investigated in the present study, but considering that the lectin and classical pathways differ only in the first step of the cascade [21], the inhibitors of the classical pathway could be equally effective on the lectin pathway. Conversely, the existence of inhibitors specific to the lectin pathway cannot be discarded.

Peritrophins from the scabies mite *Sarcoptes scabiei* seem capable to bind to the MBL from the lectin complement pathway and trigger the complement cascade inside the gut [37]. Such a mechanism could act as a kind of complement scavenger and may be involved in midgut protection. It is possible that something similar could be, at least in part, responsible for midgut protection in *L. longipalpis*. According this hypothesis, a previous assemblage of the peritrophic matrix is not required assuming that peritrophins can be capable to trigger the lectin pathway by itself.

Because salivary complement inhibitors are released at the bite site, they may be involved in the success of *L. infantum* transmission from the vector to the vertebrate hosts or even from infected reservoirs to sand flies. Phlebotomine sand flies are called pool feeders because their inflexible and short mouthparts cannot be used to search and cannulate blood vessels under the host skin, unlike the triatomine bugs [38]. Sand flies insert their mouthparts into the skin and disrupt tissue and vessels, producing a pool containing blood and tissue debris to ingest [13]. In the vertebrate hosts, amastigotes of *L. infantum* live inside phagocytic cells such as macrophages, and most amastigotes are encountered in macrophages localized outside the blood vessels, in the interstitium of internal organs or in the skin. The concentration of the complement components in the interstitium is considerably lower than in the blood [39]. If an infected macrophage undergoes rupture in the interstitium and the amastigotes are released, despite being relatively sensitive to the complement [40], they are not killed and can be promptly phagocytised by other macrophages. During its life cycle, *L. infantum* parasites only enter directly into contact with the high concentration of complement present in the blood on vector biting occasions, when metacyclic promastigotes are being transmitted from infected sand flies to the vertebrate host or when amastigotes are being ingested from an infected reservoir. The disruption of infected macrophages at the bite site or even along the insect gut exposes the amastigotes to a high concentration of complement components that are normally present in the circulating blood. In this context, it is possible that complement inhibitors from the vectors play an important role in promoting parasite survival. 

In fact, it has been demonstrated that sera from humans, dogs and rodents are lethal for promastigote forms of *Leishmania* [41]. Accordingly, it was also demonstrated that human classical pathway activation is responsible for a significant percentage of *L. donovani* promastigote death [42] and that approximately 90% of complement activation by *L. infantum* promastigotes is performed via the classical pathway [43]. 

**Figure 6:**
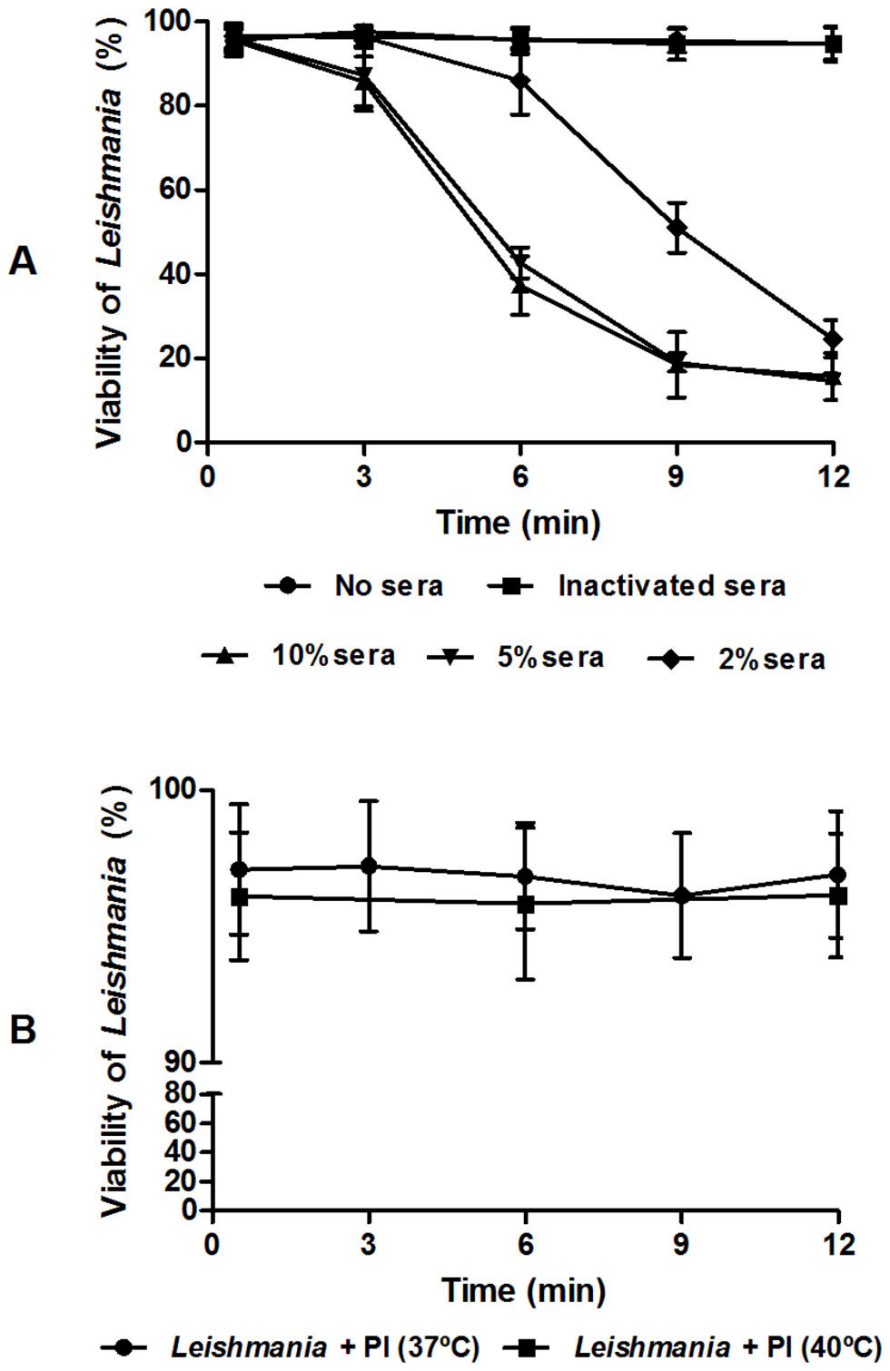
Kinetic lysis of *L. infantum* promastigotes by chicken sera. Effect of different concentrations of chicken sera (**A**) and different incubation temperatures (**B**) on the viability of *L. Infantum* promastigotes. The results are expressed as the mean of the percentage of live cells ± SD. Three replicates were performed for each experiment.

 A recent study demonstrated that chickens do not sustain *L. infantum* infections because the authors failed to experimentally infect domestic chickens with subcutaneous inocula of promastigote forms [9]. None of the inoculated animals presented clinical signs of visceral leishmaniasis, seroconversion or positive *Leishmania* results in the tissues cultures. However, the mechanism through which chickens eliminated the parasites remained unclear. In trying to understand how chickens eliminate *Leishmania* parasites, we performed kinetic lysis assays in which promastigotes were incubated with different concentrations of fresh chicken sera and assessed the viability of the parasites over time. We observed that the serum was responsible for significant parasite deaths (Figure 6A). After 12 minutes of incubation, even the lowest concentration of serum (2%) was capable of killing approximately 80% of the parasites. When inactivated serum was used, parasite deaths were prevented. This treatment confirms that parasite death could be a consequence of complement activation. 

*Leishmania* susceptibility to the chicken complement was observed in the present study and contrasts recently published results [44]. In those experiments, *L. longipalpis* were artificially fed with chicken blood containing the amastigotes of *L. mexicana*. When the females were dissected, promastigotes were present inside the midgut in more than 90% of the flies six days post-blood meal. The authors concluded that there is nothing in chicken blood that kills *Leishmania*. However, the blood used in their experiments was diluted in Alsever's solution, which contains citrate as anticlotting agent. Because citrate chelates Ca^2+^ ions, which are essential for classical pathway activation, we concluded that the classical pathway was inactive during those experiments. 

In support of our results, it was already demonstrated that a second chicken blood meal taken 24 hours post-infection by *Leishmania*-infected sand flies was responsible for 100% of parasite elimination [45]. The authors did not conclude the cause of parasite death, but based on our data, we can suggest that death was caused by complement system activation. Chickens are also refractory to *Trypanosoma cruzi*, the causative agent of Chagas Disease, a protozoan parasite that belongs to the same family as *Leishmania* (Trypanosomatidae). This phenomenon occurs because of the lytic effect of the avian complement system on trypomastigote forms of *T. cruzi*, which become undetectable in blood after four minutes following chicken inoculation [46]. In the same study the authors demonstrated that 1 µl of chicken sera is capable of lysing approximately 1-3 x 10^7^ *T. cruzi* parasites.

**Figure 7:**
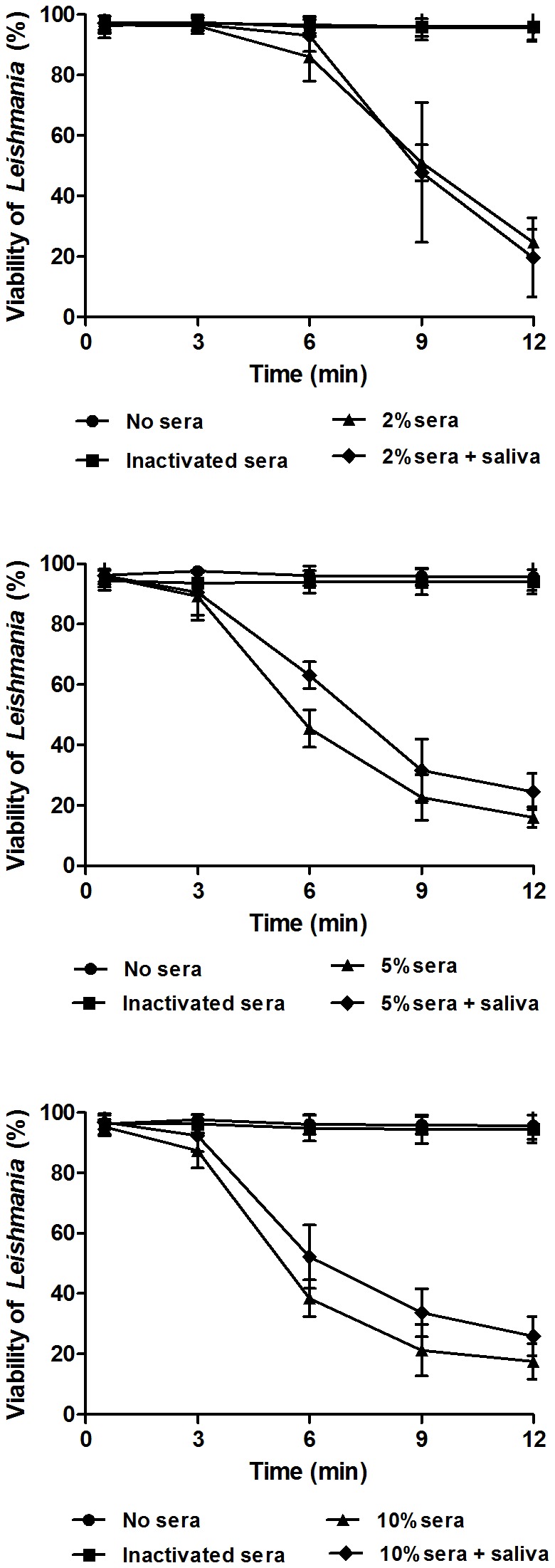
Kinetic lysis of *L. infantum* promastigotes by different concentrations of chicken sera in the presence and absence of *L. longipalpis* saliva. The results are expressed as the mean of the percentage of live cells ± SD. Three replicates were performed for each experiment.

 We recently observed that amounts of saliva equivalent to eight salivary glands of *L. longipalpis* is capable of protecting *L. infantum* promastigotes from death caused by the human complement system (unpublished data). To investigate whether the saliva could protect the parasites against the chicken complement system, we performed a kinetic lysis assay in the presence of eight salivary glands (Figure 7). No protection against the avian complement system was observed, even when the less concentrated sera (2%) was used (P = 0.9871), confirming our previous results as presented in Figure 6. 

It was suggested that the high body temperature of chickens (approximately 40°C) could also be responsible for *Leishmania* clearance [6,10]. To determine whether the promastigotes were affected by the temperature during the incubation time, we incubated *Leishmania* promastigotes with propidium iodide, but without sera at 37 °C and at 40°C, and followed up on the parasite viability for 12 minutes. No significant differences in viability were observed during this time (P = 0.5161) (Figure 6B). *Leishmania* does not survive for long periods at 40°C, but in our experiments, the complement was the unique host component responsible for *Leishmania* destruction, at least in the first 12 minutes of exposure. 

## Conclusions

The classical pathways of the species of interest (dogs, guinea pigs, rats and chickens) were inhibited with different degrees of efficiency by saliva or the intestinal content from unfed *L. longipalpis* females, but the alternative pathway was not efficiently inhibited. Although not investigated in the present study, lectin pathway inhibitors and other putative complement inhibitors linked to the membranes of the enterocytes could be present and would be co-responsible for effective midgut protection.

The complement of chickens is very efficient at killing promastigote forms of *Leishmania infantum*, even at 2%. Saliva from *L. longipalpis* was not protective because it very inefficiently inhibits the avian complement system. This behaviour is most likely part of the explanation for why chickens are not susceptible to leishmaniasis.
